# A novel urethra compression technique using Turkish continence device for male urinary incontinence

**DOI:** 10.3906/sag-1907-5

**Published:** 2020-08-26

**Authors:** Öner ODABAŞ, İrem Gül SANCAK, Yusuf KASAP, Zerrin MAHMUT, Erkan ÖLÇÜCÜOĞLU, Sedat TAŞTEMUR, Neslihan ZENGİN

**Affiliations:** 1 Urology Clinic, Ankara Bilkent City Hospital, University of Health Sciences, Ankara Turkey; 2 Surgery Department, Faculty of Veterinary Medicine, Ankara University, Ankara Turkey; 3 Pathology Laboratory,Ankara Bilkent City Hospital, University of Health Sciences, Ankara Turkey

**Keywords:** Male urinary incontinence, treatment, novel compression technique, experimental study, Turkish Continence Device

## Abstract

**Background/aim:**

The male sling operation and artificial urinary sphincter implantation are common methods for treating urinary incontinence. However, there are some drawbacks to these methods such as infection, urethral erosion, pain, inefficiency, and the technical difficulty of the operations. Here we describe a new device we have named the Turkish Continence Device (TCD) which has advantages over these other methods. The aim of this study was perform experiments with the TCD prototype in vivo and ex vivo to determine efficiency, convenience of implantation, and negative effects.

**Materials and methods:**

We implanted the prototype device in male goats and sheep, compressing the posterior urethra, and then fixed it by sutures on the lateral sides of the cavernosal bodies, bilaterally. Then we recorded urodynamic findings and performed urinary imaging. Additionally we measured urethral closure pressure ex vivo.

**Results:**

The balloon volume for efficient urethral closure pressure using the new device was under 1 mL. It compressed the urethra towards the corpus cavernosum perfectly, because the wings of the prototype device are fixed near the tunica of the cavernosal bodies on each side.

**Conclusion:**

A smaller device with smaller arms/wings would be efficient for obtaining enough pressure on the urethra. Additionally, the technique for implanting the device is very simple and would likely be learned quickly.

## 1. Introduction

With the increasing numbers of radical prostatectomies, male urinary incontinence has become common. Multicenter studies and prostate cancer databases show that after radical prostatectomy 1% to 40% of patients complain of persistent urinary incontinence [1–3]. This large variation may be based on the influence of the interviewing physician and the lack of standardized description of postprostatectomy incontinence. Iatrogenic-induced sphincter incompetence is the cause of postoperative stress incontinence in 95% of cases [4]. Many devices and techniques for treatment of urinary incontinence have been identified. But none are completely efficient, as there are drawbacks involving each of them such as infection, urethral erosion, serious pain, inefficiency, and the technical difficulty of the operations.

The aim of this experimental study is to develop a novel prototype device (TCD) for achieving urinary continence by compressing the male urethra towards the corpus cavernosum.

## 2. Materials and methods

**Figure 2:**
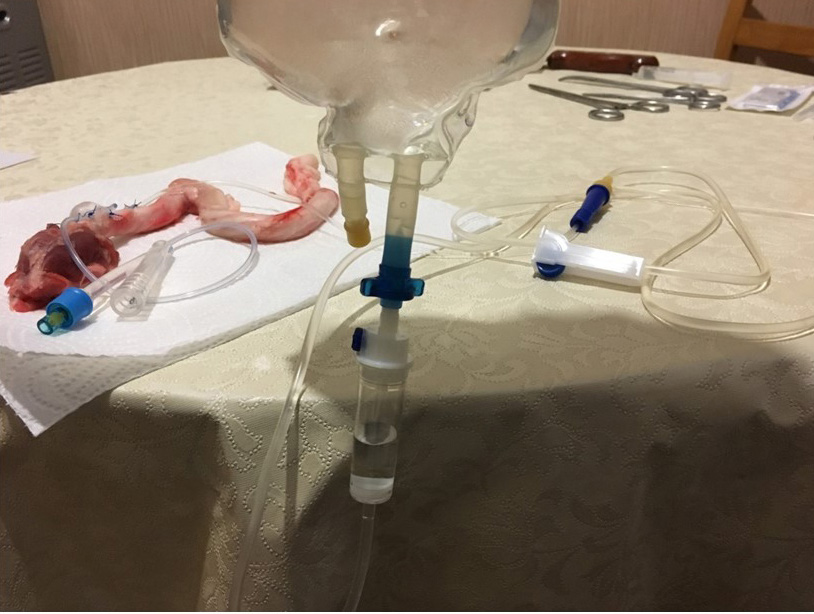
Measuring urethral closure pressure at different volumes of TCD’s balloon.

We used 3 male goats and 3 male sheep for the study, and each animal was approximately 12 months old. Before the study elaborating the anatomy of the animals, we performed a cadaveric dissection of the penis and urethra of these species. After that we excised the urethra and penis as a block. The proximal urethra was chosen for modeling and measuring the urethral pressure produced by the TCD. We studied urethral closure pressures on the apparatus using the excised penis–urethra specimen with the prototype device implanted on it. We created the apparatus by placing a prototype device on the proximal urethra by suturing to the lateral surfaces of the tunica albuginea of the cavernosal bodies bilaterally with two sutures on each side (Figure 1). The prototype of the novel device included a Foley catheter (6F or 8F) covered all around with Prolene mesh. To avoid displacement of the Foley balloon on the urethra, the mesh leaves were braided using Prolene stitches around the Foley balloon. Two Prolene wings were left bilaterally to fix the device through suturing on the tunica of the cavernosal body (Figure 1). Then, we inflated the Foley balloon with saline (0.3–1.5 mL) until it stretched. We inserted a Nelaton catheter from the distal transection of the urethra, and this catheter was connected to a saline bag via a serum set to measure the urethral pressure produced by the external compression of the TCD (Figure 2). When maximal stretch of the balloon is reached, fluid flowing from the saline bag is stopped. Then we shrank the balloon gradually and measured urethral closure pressure (UCP). 

**Figure:**
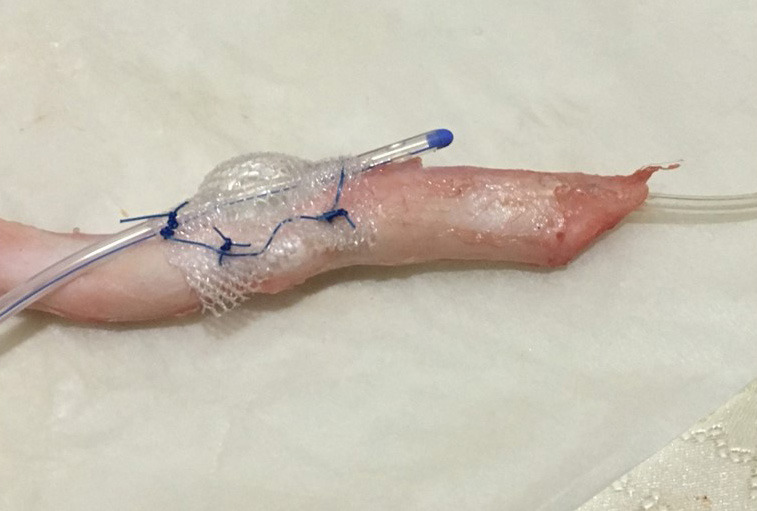
Prototype of novel devices includes for a Foley catheter (6F or 8F) which its balloon covered with prolene mesh. Two prolene wings fixed by suturing on cavernosal body’s tunica bilaterally.

After the postmortem examination of the animals, we implanted the novel prototype devices in live animals. We operated on three male sheep and three male goats in order to implant the TCD model. Before the operations we administered xylazine hydrochloride (0.2 mg/kg) intravenously for premedication followed by ketamine (17.5 mg/kg) intravenously for general anesthesia.

Under general anesthesia, the perineal area was shaved and cleaned at the decubitus position. After painting the perineal region with Batticon, we made a vertical incision on the posterior part of the penile structures. Then we located and isolated the cavernosal and spongiosal bodies. We made an incision in the corpus spongiosum which is thin and very adherent to the urethra in sheep and goats. After incising the posterior urethra, we indwelled a 6F or 8F Nelaton catheter for filling cystometry. It was very difficult to attach the catheter transurethrally in rams and goats without administering a muscle relaxant.

We implanted TCD prototypes on the posterior urethra by fixing them to the tunica albuginea of the cavernosal bodies on each lateral side in all animals. The opposite side of the Foley catheter has two tips; one is for balloon inflation/deflation, and the other is for urine drainage. We cut the tab of the urine drainage channel to facilitate passing the catheter along the inner scrotal wall and out of an incision on the lateral scrotal wall. We proposed to inflate/deflate the balloon from this tip in order to arrange urethral pressure after the operation. After implanting the device and removing the inflating/deflating channel tip of the Foley catheter from the body, we finished the operation by closing the urethrostomy and skin incisions. 

We inflated the Foley balloon with 0.3–1.5 mL of saline in order to stretch it very tightly. After closing the incisions, all animals were clothed to check for wetting with urine. We wondered whether the animals would urinate or not and injected a diuretic (Furosemide 2 mg/kg) in order to observe this more quickly.

On the 7th day, a ram and a goat underwent an imaging study to evaluate the degree and effect of urethral obstruction. We performed intravenous nephro-pyelography and retrograde urethrography.

We fed all the animals for 1 month and then sacrificed them. We excised the posterior urethra, including the implanted TCD prototype, for pathologic investigation.

## 3. Results 

After inserting the Nelaton catheter into the urethra in the prototype device complex (apparatus 1), we measured UCP. We observed that a little balloon volume was enough to obtain efficient urethral closure pressure. The necessary balloon volume for efficient UCP is under 1 mL which shows that continence would be achieved in incontinent men using very small devices. We repeated this measurement procedure on apparatus 2 and reported all UCP values at different volumes of Foley balloon in Table 1. 

**Table 1:** Urethral closure pressures at different volumes in the baloon on apparatuses 1 and 2.

Saline volume in theballoon (ml)	Apparatus 1 urethral closure pressure (cmH2O)	Apparatus 2 urethral closure pressure (cmH2O)
1.5 ml	160	145
1.2 ml	115	95
0.9 ml	88	65
0.6 ml	56	34
0.3 ml	28	16
0 ml	12	10

We were able to perform an urodynamic study intraoperatively on two rams and two goats. The findings of these studies are reported in Table 2. 

**Table 2:** Bladder pressures at different bladder volumes in 4 subjects.Goat 1Goat 2Ram 1Ram 2Detrusor pressure of full bladder (cm H2O)40306030Bladder capasity (ml)48042025040

	Goat 1	Goat 2	Ram 1	Ram 2
Detrusor pressure of full bladder (cm H2O)	40	30	60	30
Bladder capasity (ml)	480	420	250	40

We determined that the TCD prototype did not cause complete obstruction because the clothes on all of the animals became wet.

**Figure 3:**
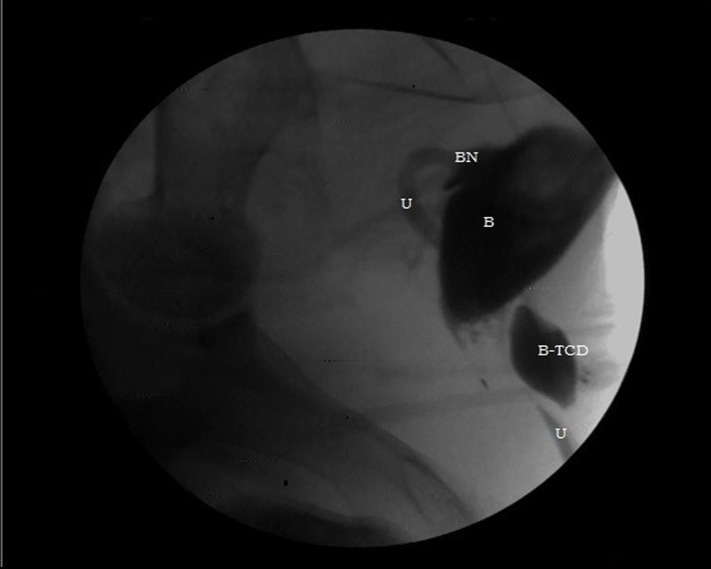
Urethrography that shows balloon impression on urethra and effect of partial urethral obstruction (B: Bladder, BN: Bladder neck, U: Urethra, B-TCD: Balloon of TCD).

We could not establish any pathology on the renal and vesicourethral images. Then we checked a Foley balloon and saw that it had shrunk. At least the half of the saline that was injected into the balloon during the operation had emptied. We reinjected saline mixed with a radiopaque substance then performed urethrography and saw the balloon impression on the urethra and the effects of a partial urethral obstruction (Figure 3). We reinjected the previous amount of saline (0,3-1,5 mL) into the Foley balloon fırt each animals.

**Figure 4:**
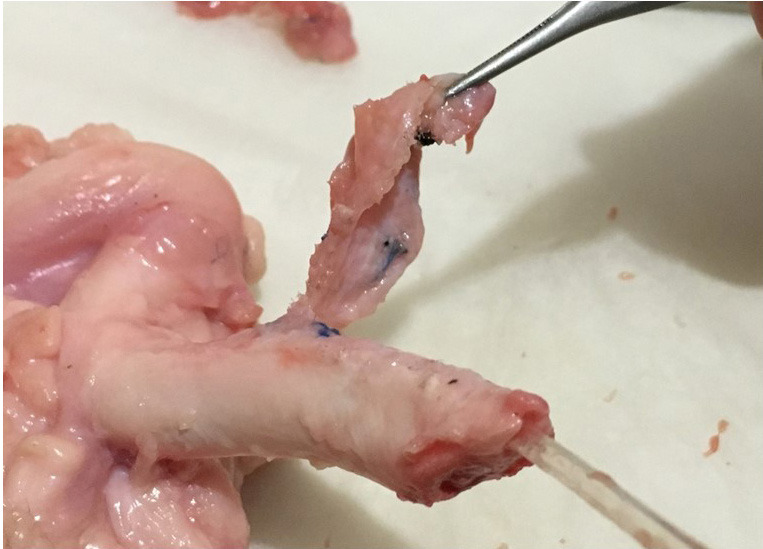
Dissecting and separating of prolene mesh from urethral wall shows that there were not any findings of urethral erosion or damage.

**Figure 5:**
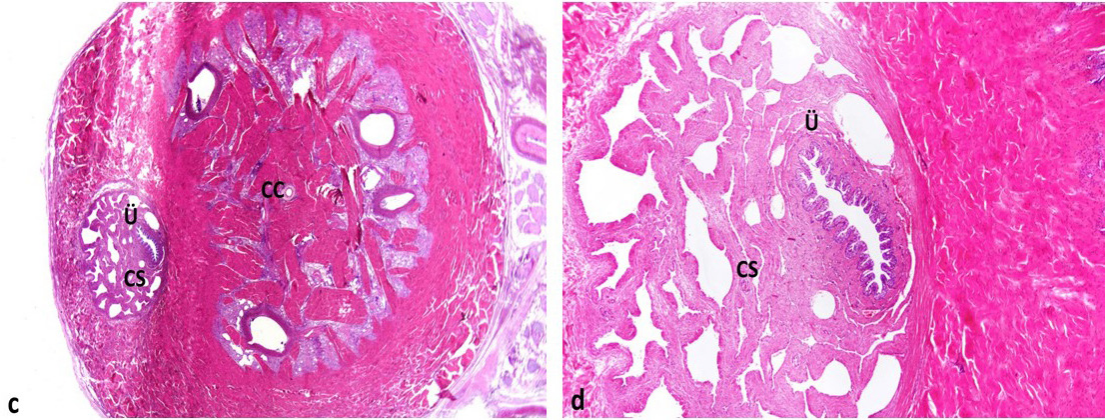
Microscopic examination confirmed that there were not any pathologic changes in urethra and corpus spongiosum (Ü: Urethra, CS: Corpus Spongiosum, CC: Corpus Cavernosum) are normal on section at TCD implanted level. There are not any pathologic changes due to TCD.

When we sacrificed the animals and checked the Foley catheter balloons we saw that they had shrunk again. Macroscopically, Prolene mesh was embedded in the tissues surrounding the urethra. It was dissected and the urethral wall was isolated. There were no findings of urethral erosion or damage due to the Prolene mesh (Figure 4). Microscopic examination confirmed these findings; there were no microscopic pathologic changes in the urethra and corpus spongiosum (Figure 5).

## 4. Discussion

The first artificial urinary sphincter was introduced in 1973 to treat urinary incontinence [5]. The AUS 800, which is the fifth generation model, was created in 1983 [6]. Recently, new devices have been developed to eliminate some of the disadvantages of the AUS 800. 

In an effort to improve the efficacy of AUS and continence, tandem cuffs have been used with idea that increasing the resistance over a greater area may improve continence. Brito et al. first described a successful tandem cuff placement, achieving 95% success [7]. Using two cuffs would lead to increased resistance to leakage without increasing pressure on a single segment of the urethra. However, a subsequent, longer follow-up demonstrated a higher risk of complications when using tandem cuffs [8].

In patients with recurrent incontinence secondary to erosion, subcuff urethral atrophy, inadequate urethral coaptation, or, for patients undergoing revisions where more proximal placement could not be achieved, transcorporal cuff placement may improve continence [9]. In a prospective series of transcorporal AUS placements, dry or socially continent rates were reported to be 76% at a median follow-up of 20 months [10].

There is a concern that transcorporal cuff placement affects erectile dysfunction. However, most patients already have some degree of erectile dysfunction at baseline due to prostate cancer treatment [10–12]. A small series reported that the majority of patients maintain their erectile function even after transcorporal cuff placement (5/6, 83%) [10].

Transcorporal cuff placement is the only described method in which the corpus cavernosum serves a function in incontinence treatment. In our study, we also used the corpus cavernosum’s tunica albugnea for fixing the TCD; however, our method did not include dissecting the cavernosal body as in transcorporal cuff placement. For this reason, this new method is less invasive and we expect that it will not have any effect on erectile function.

The flow secure sphincter was manufactured with a supplemental reservoir to relieve stress pressure during intraabdominal pressure increase. During the bladder filling phase at low pressure, the pressure-regulating reservoir keeps the bulbous urethra closed. When intraabdominal pressure rises, the stress relief balloon ensures additional pressure to the cuff to secure continence [13–15].

The ZSI 375 (Zephyr Surgical Implants, Geneva, Switzerland) has been developed recently to facilitate AUS implantation1Zephyr Surgical Implants (2016). ZSI 375 **Artificial Urinary Sphincter** [online]. Website u2985 [accessed 15 September 2016].. The ZSI 375 consists of one piece and is made up of 2 parts connected by tubing. One of these parts is an adjustable cuff formed to fit around the urethra. The other part is a pressure-regulating tank and pump that is placed in the scrotum [16,17].

Another improvement in the artificial urinary sphincter area is a novel, remotely-controlled sphincter [18]. It was developed using an AMS 800 to replace the pump. The new electronic pump has been designed to be as small as possible to facilitate implantation and compatibility with the existing AMS 800 tubing, cuff, and balloon. The device is completely wireless and, when tested on a fresh pig’s bladder, continence was achieved.

A novel artificial urinary sphincter tested in a canine model is the tape mechanical occlusive device (TMOD) [19]. The TMOD is a one-piece device for urinary incontinence that utilizes a spring loaded mechanism to apply constructive circumferential pressure on the urethra. Malaeb et al. tested the functionality and biocompatibility of the device in canines and examined the sizing and occlusive efficiency in human cadavers.

An animal model was also used to improve a new electromechanical artificial urinary sphincter (emAUS) [20]. The emAUS consists of two parts; a contractile unit, including two or more urethral cuffs that apply synchronized sequential compression to the urethra, which is implanted intracorporeally, and an electronic board which controls it extracorporeally. The fibers of each contractile unit are composed of nitinol, a metal alloy of nickel and titanium. When the artificial muscles forming each contractile unit are relaxed, the urethra opens. The animal study, which was performed on male sheep, showed that the emAUS can provide continence. This new, electronically controlled sequential alternating compression mechanism can circumvent damage to urethral vascularity at least up to 3 months after implantation. 

Fabio Vilar improved the periurethral constrictor (PUC) device in 1996 [21]. The PUC is a one-piece, two-part device. It is comprised of a constrictor cuff linked to a valve, which is elliptical in shape, by a 20 cm silicone tube. The valve is placed in a space accessible by percutaneous puncture, and the system works hydraulically through the injection of sterile saline solution.

A limited number of studies with controversial results have been published regarding use of PUC in postprostatectomic urinary incontinence (PPUI). In one study 30 patients with PPUI were treated with PUC implantation. In 22 patients (73.3%), good continence was achieved [22]. In another study performed by Introini et al., 66 patients with urinary incontinence following radical prostatectomy were treated by PUC implantation. Continence was recovered completely in 49 cases (79%), partially in 9 (15%) cases, and remained unchanged in 4 (6%). In 4 cases (6%) the device was removed because of infection/periurethral erosion [23].

Lima et al. reported a study with a very high device removal rate 41.7% [24]. The most frequent complication was urethral erosion (15 patients, 26.78%). Other complications were mechanical malfunctions in 5, urethral stenosis in 3, urinary fistula in 2, infection in 2, and persistent urinary tract infection in 1 case. The overall success rate was 30.35%.

Male sling procedures are the most commonly used methods for the treatment of urinary incontinence. Tapes are available, and the sling mechanism is based on the concept of passive external urethral compression. Various sling systems have been described. The Argus system is an adjustable sling including a silicone cushion, two silicone columns, and silicone rings/washers. It allows for implant adjustment and regulation of the desired tension. There are two techniques for implantation; the retro-pubic approach and the transobturator approach (Argus T).

Hübner reported a series of 101 male patients treated with the Argus sling for SUI [25]. Of these patients, 22 (21.8%) were treated with RT for local recurrence of prostate cancer. After Argus implantation, adjustment was necessary in 39 cases (38.6%). The second adjustment to the sling tension was made in 7 patients. The third and fourth adjustments were made in 3 patients and 1 patient, respectively. The median follow up was 2.2 years and 80/101 (79.2%), and patients were considered dry with a pad test of 0–1 g. Sixteen patients (15.8%) had complications requiring device removal due to urethral erosion or infection after a mean of 371 days (range: 20–1260).

The other male sling type is the four-arm I-STOP transobturator male sling (TOMS) (CL Medical), which is a monofilament polypropylene device. The dimensions are 45 × 1.4 cm with a 2.8 cm central part that is placed over the urethra. Grise et al. reported a multicenter prospective study including 122 patients with PPUI (94.9% radical prostatectomy) [26]. At 12 months, only 69 patients were available and 60 (87%) of them had improvement in the number of pads used daily; 41, 14, and 5 patients reported 0, 1, and >1 pad daily (PPD), respectively. The only complication described was wounding of the corpus cavernosum (4% of the patients).

The AdVance transobturator male sling (AMS, Minnetonka, MN, USA) is also a polypropylene monofilament mesh placed retro-urethrally by passing tapes bilaterally through the obturator fossa. The AdVance male sling essentially realigns the anatomy of the urethral sphincter complex towards the normal configuration. Rehder et al. reported a multiinstitutional study with 156 patients treated with the AdVance male sling for SUI [27]. Patients were considered cured if no pad or 1 dry pad for security were used and if there was a reduction in daily pad usage of 50%. All other situations were considered failure. After implantation of the AdVance sling, pad usage was significantly decreased compared with baseline (P < 0.0001) at 1 and 3 years. In the 1st year, 76.9% of patients were classified as cured or improved, and this improvement was 75.7% at the 3rd year. In total 109 complications were registered which were mostly Dindo grade 1 (90 patients). The most common complication was mild perineal pain. One sling device was explanted because of symphysitis. The transient urinary retention rate was 9%.

The adjustable transobturator hydraulic male system (ATOMS, AMI, Vienna, Austria) shares some similarity with our device (TCD). The ATOMS has two components; the adjustable cushion with mesh tapes suspending it using the transobturator approach and the implantable titanium port for adjusting the tension of the cushion and pressure on the urethra [28]. Honda et al. reported the first results of ATOMS implantation [29]. The most common indications for placement of this device were SUI after RP (92/99 patients) and failure of previous continence device implantation surgeries (34/99). The mean pad use decreased from 7.1 to 1.3 pads daily (P < 0.001), and 63% patients were classified dry (0 pad: <10 mL at 24 h pad test) and 29% were improved (1–2 pads: 10–40 mL daily). The overall success rate was 92%. The most frequently (68.7%) reported complications were transient pain or numbness referred to the perineum, scrotum, or thighs which required use of nonopioid analgesics for three to four weeks. Wound infection at the site of the titanium port (leading to complete explantation of the device) occurred in 4/99 patients. 

The TCD is not a real artificial urinary sphincter, and it is different from sling materials and methods as well. In male sling methods, the posterior bulbous urethra is hung by passing the arms of the sling material through the obturator foramen or retro-pubic space bilaterally. The TCD does not require long arms/tapes for fixation. Short wings are enough to affix it to the cavernosal tunica on both sides. Furthermore, it will be adjustable in humans with the inflation–deflation port placed under the scrotal skin. The pressure on the urethra would be adjusted by changing balloon volume via injector needle insertion in the inflation–deflation port through the punctured scrotal skin. 

In conclusion, the TCD is much smaller than other devices because it does not require long arms or tapes. It provides sufficient pressure for continence because it sitting exactly on the urethra. We use the cavernosal fascia for holding and fixing the TCD instead of passing the tapes along retro-pubic or transobturator routes. Because the device is small and uses the least invasive implantation technique, we estimate complications such as pain, infection, or urethral erosion will be minimal. Additionally, the surgical procedure is simpler, the implantation learning curve may be shorter, and peroperative complications (organ, vessel, or nerve injury) may be lower than in all other continence device operations. 

## Acknowledgment

This experimental study was performed in the surgery clinic of the Faculty of Veterinary Medicine, Ankara University, Ankara, Turkey.

## Animal welfare

We affirm that all experiments were performed in compliance with the relevant laws and/or institutional guidelines. The study was approved by the animal ethics committee, Faculty of Veterinary Medicine, Ankara University (2015-20-219 and 2015-20-220).
